# Stimulus-Responsive Shrinkage in Electrospun Membranes: Fundamentals and Control

**DOI:** 10.3390/mi12080920

**Published:** 2021-07-31

**Authors:** Feiyu Fang, Han Wang, Huaquan Wang, Wei Min Huang, Yahui Chen, Nian Cai, Xindu Chen, Xin Chen

**Affiliations:** 1 Guangdong Provincial Key Laboratory of Micro-Nano Manufacturing Technology and Equipment, School of Electromechanical Engineering, Guangdong University of Technology, Guangzhou 510006, China; feiyu93@foxmail.com (F.F.); chenxindu@gdut.edu.cn (X.C.); chenx@gdut.edu.cn (X.C.); 2 State Key Laboratory of Precision Electronic Manufacturing Technology and Equipment, School of Electromechanical Engineering, Guangdong University of Technology, Guangzhou 510006, China; 3 China Resources Cement Technology Research and Development Co., Ltd., Guangzhou 510460, China; whq931@163.com; 4 School of Mechanical and Aerospace Engineering, Nanyang Technological University, 50 Nanyang Avenue, Singapore 639798, Singapore; 5 School of Physical Science and Technology, Soochow University, Suzhou 215006, China; chenyahui@suda.edu.cn; 6 School of Information Engineering, Guangdong University of Technology, Guangzhou 510006, China; cainian@gdut.edu.cn

**Keywords:** electrospinning, shrinkage, buckling, shape memory effect, gradient strain field

## Abstract

Shrinkage is observed in many electrospun membranes. The stretched conformation of the macromolecular chains has been proposed as the possible cause. However, so far, our understanding of the fundamentals is still qualitative and cannot provide much help in the shrinkage control. In this paper, based on the crimped fibers after stimulus-induced shrinkage, a clear evidence of buckling, the gradient pre-strain field in the cross-section of the electrospun fibers, which is the result of a gradient solidification field and a tensile force in the fibers during electrospinning, is identified as the underlying mechanism for the stimulus-induced shrinkage. Subsequently, two buckling conditions are derived. Subsequently, a series of experiments are carried out to reveal the influence of four typical processing parameters (namely, the applied voltage, solution concentration, distance between electrodes, and rotation speed of collector), which are highly relevant to the formation of the gradient pre-strain field. It is concluded that there are some different ways to achieve the required shrinkage ratios in two in-plane directions (i.e., the rotational and transverse directions of the roller collector). Some of the combinations of these parameters are more effective at achieving high uniformity than others. Hence, it is possible to optimize the processing parameters to produce high-quality membranes with well-controlled shrinkage in both in-plane directions.

## 1. Introduction

Electrospinning is a commonly used technology for producing fiber/nanofiber membranes and has been widely used in tissue engineering, filtration systems, flexible electronics, etc. [1,2,3,4,5,6]. Many electrospinning fiber membranes (e.g., polyvinyl alcohol (PVA), poly (lactic acid) (PLA), poly(lactide-co-glycolide) (PLGA), polysulfone (PSF), polyvinylpyrrolidone (PVP), poly-ε-caprolactone (PCL)) shrink under certain conditions, e.g., upon heating and/or immersing in a right solvent [4,5,7,8,9,10,11,12]. Such a kind of shrinkage may cause some serious problems (such as scaffold deformation, collapse, etc.) in tissue engineering applications, but they also can be utilized, e.g., to prepare crimped fibers and self-folding electrospun scaffolds [4,5,9,13]. Although crimped fibers can be produced via co-electrospinning of two different materials with different shrinkage ratios [13,14], in addition to a far more sophisticated system, a special nozzle is required [15]. 

The stretched conformation of the macromolecular chains has been suggested as the cause of the shrinkage of the electrospun membranes [4,5]. The network of entangled polymer chains of semidilute entangled solution is stretched by the external force. It leads to a steady high molecular orientation and is frozen due to the rapid evaporation of the solvent. This unbalanced molecular conformation recovers under the right external stimuli, so the fibers shrink. However, at present, this explanation is only at a qualitative level and cannot provide much useful guidance for controlling the shrinkage, either to avoid undesired shape change or to be harnessed in a precise manner for real engineering applications.

The shape memory effect (SME) refers to the ability of a material to recover its original shape, but only when the right stimulus is applied. Those materials with the SME are called shape memory materials (SMMs) [16,17]. Depending on the type of SMM, the stimuli that can active the SME include heat (thermo-responsive), chemical (including water, chemo-responsive), light (photo-responsive) and magnetic field (magneto-responsive). While the SME is limited to some particular alloys (called shape memory alloys, SMAs), most polymers intrinsically have the heating-responsive SME and chemo-responsive SME [18]. For a lot of engineering polymers, some chemicals (including water and even moisture) may act as a plasticizer to reduce their glass transition temperature (T_g_) even below the room temperature [19]. Consequently, their shape recovery can be activated by immersing in the right chemical without the heating process [20]. In addition to the plasticizing effect, which is generic for the glass transition-based SME in polymers, some other working mechanisms are able to induce the chemo-responsive SME in some special types of polymers [21,22].

A typical shape memory cycle includes two processes [23]. The first process (called programming) is to fix the temporary shape, and the second process (called recovery) is to apply the right stimulus to active the SME. The programming process is required to store the elastic energy in the elastic part of SMPs [18]. The stored elastic energy provides the driving force for shape recovery in the step. The programming process may be integrated in the material fabrication/processing part [24].

The purpose of this paper is twofold. One is to reveal the fundamentals behind the shrinkage of the electrospun membranes, including the underlying mechanism and the critical buckling conditions. The other is to investigate the feasibility of controlling the shrinkage in two in-plane directions of the electrospun membranes via a series of experiments, in which four key processing parameters, identified based on the understanding of the fundamentals, are chosen as the variables.

## 2. Materials and Method

### 2.1. Materials and Preparation

The thermoplastic polyurethane (TPU) MM5520 (pellets) used for electrospinning was purchased from SMP Technologies Inc. (Tokyo, Japan), with a nominal glass transition temperature (T_g_) of 55 °C. As reported in [25], after being immersed in water or ethanol, the T_g_ of this TPU can be lowered by 30 °C, due to the plasticizing effect of the absorbed bound water or ethanol on the hydrogen bonding [26]. Hence, the SME of this TPU can be activated either by heating to above its T_g_ or by wetting in ethanol.

N,N-dimethylformamide (DMF), ethanol, and acetone were purchased from Sinopharm Chemical Reagent Co., Ltd. (Shanghai, China). DMF was mixed with acetone in a volumetric ratio of 1:1. After that, TPU pellets were added into it for different concentrations varying from 10 wt % to 25 wt %. Subsequently, magnetic stirring was applied at room temperature (≈25 °C) for 24 h.

### 2.2. Fabrication of Electrospun Membranes

**Figure 1:**
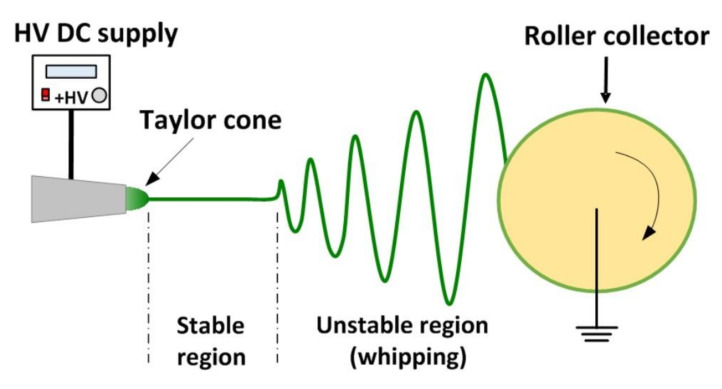
Schematic illustration of electrospinning system.

Figure 1 schematically illustrates the electrospinning system used in the course of this study. The prepared solution for electrospinning was placed into a syringe equipped with a 22 G dispensing nozzle (inner diameter 420 μm and external diameter 720 μm), and a pump (Pump 11 Elite, Harvard Inc., Holliston, MA, USA) was used to feed 5 mL of solution at a constant speed for membrane fabrication. A high-voltage supply (DW-P303, Tianjin Dongwen Inc., Tianjin, China) was applied to the nozzle. A speed-adjustable rotating cylinder (with a diameter of 10 cm covered by aluminum foil and width of 5 cm) served as the collector and was connected to the negative electrode. The applied voltage (kV), solution concentration (wt %), distance between electrodes (cm), rotation speed of the collector (rpm/min), and solution flow rate (mL/h) were taken as the control parameters in the fabrication process. The electrospinning experiments were performed at room temperature without any special control in humidity. Refer to Table S1 in the Supplementary Materials for the processing parameters of each group.

### 2.3. Shrinkage Test

**Figure 2:**
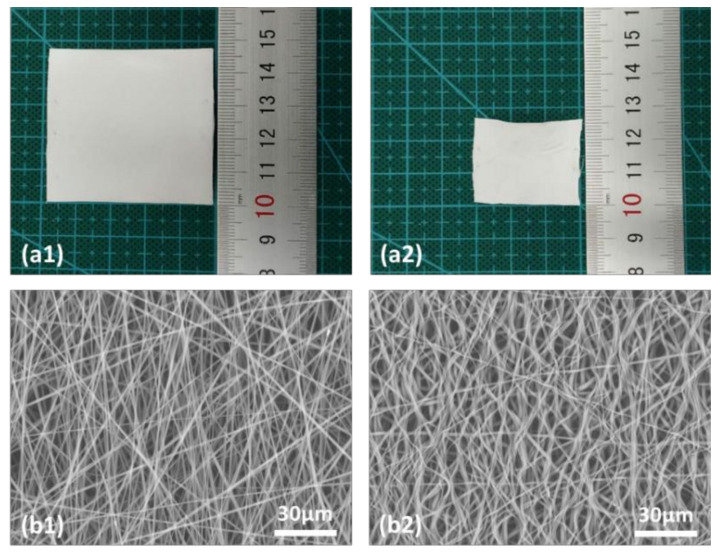
(**a**) Typical sample (applied voltage: 10 kV; solution concentration: 15%; distance between electrodes: 10 cm; speed of collector: 700 rpm/min; solution flow rate: 1.2 mL/h) before (**a1**) and after (**a2**) ethanol wetting. (**b**) SEM images before (**b1**) and after (**b2**) wetting.

As-fabricated TPU membranes were taken out of the roller collector. Small samples with a size of about 4.8 cm × 4.8 cm were cut from the membranes. It is noticed that when the membranes were taken out of the roller collector, about 4% shrinkage in both the rotational direction and the transverse direction was observed. Ethanol (concentration: 99%) was used to activate the shrinkage of TPU samples. To avoid tangling due to rapid and severe shrinking, TPU samples were wetting by ethanol droplets from one side to the other side gradually. Refer to Video S1 in Supplementary Materials for a video of the process of the controlled ethanol wetting of membrane. TPU samples were fully wetted for two to three min, until no further deformation was observed. The change in length (D_i_, i = x and y, where x is the rotational direction of the roller and y is the transverse direction, respectively) was recorded (Figure 2a), and the shrinkage ratios in both the rotational direction of collector and the transverse direction were calculated as D_i_/L_i_,_0_ (%), where L_i,0_ is the original length before wetting.

### 2.4. Morphology Characterization

The morphology of the samples before and after ethanol wetting was revealed by a scanning electron microscope (SEM; TM3030, Hitachi Inc., Tokyo, Japan). The distribution of the fiber alignment of the as-fabricated membrane was calculated using Image J software.

## 3. Results and Discussion

### 3.1. Membrane Shrinkage and Fiber Crimp

Figure 2b presents typical SEM images before and after ethanol wetting. Refer to Figures S1–S3 in Supplementary for more SEM results of this TPU, another TPU, and poly (lactic acid) (PLA), respectively.

The SEM images of the as-fabricated membrane samples and the distribution of the fiber alignment in three samples fabricated at different rotation speeds are presented in Figures S4 and S5 in the Supplementary Materials. It appears that the rotation speed of the roller, in particular a higher rotation speed (e.g., 700 rpm), does cause more alignment of the fibers, which implies a kind of straightening effect when the fibers are collected on the roller.

About 4% shrinkage in both directions as we observed during taking the membranes out of the roller does not induce any apparent crimping in the fibers. This finding implies that the shrinkage, which is much smaller when compared with the shrinkage induced by ethanol, is mostly elastic deformation and uniform (i.e., without buckling) in the whole fibers. De-swelling during further evaporation of the solvent after the fibers have been collected by the roller should be the major source for the tensile force, while the straightening effect may have limited the contribution to the tensile force.

### 3.2. Underlying Mechanism behind Membrane Shrinkage

Recall Figure 1. In the electrospinning process, a high voltage is applied on the needle to form a high-voltage electric field. Due to the accumulation of electric charge, the droplet hanging on the needle is stretched into an elongated cone shape, which is known as a Taylor cone [27]. When the voltage reaches a critical value, the electric field causes the charged liquid to overcome the surface tension of the solution and to release an elongated jet from the tip of the cone. Due to the combined action of the electric field force and the Coulomb repulsive force, the charged thin jet becomes unstable and whipping. In the meantime, the diameter of the jet decreases rapidly until the jet becomes fiber and collected by the collector [15,28]. If a high-speed rotating drum is used as the collector, a tensile force is applied on the fiber. In this whole process, the solvent evaporates and the liquid jet gradually becomes thin solid fiber.

**Figure 3:**
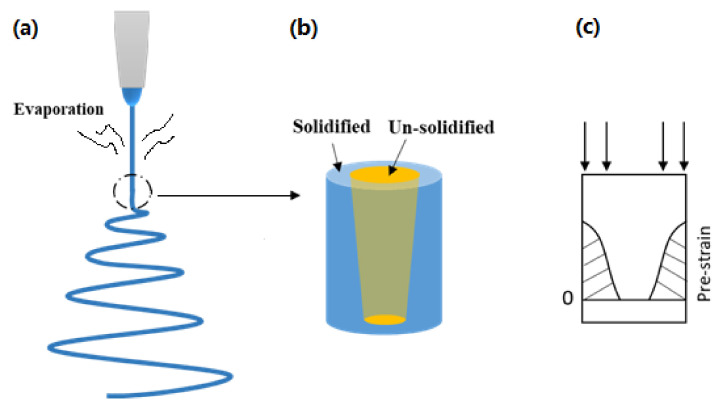
Illustration of electrospinning (**a**), gradual solidifying (**b**), and resulted pre-strain field (**c**).

The evaporation of the solvent is inhomogeneous in the radial direction of the jet/fiber [29,30]. As an example, Figure 3a,b reveal that the solidification/drying of the jet starts from the surface of the jet (shell) and gradually moves into the core. The solidified shell (only partially solidified) is stretched in an elastic–plastic manner, while the inner unsolidified core is able to flow easily, which results in a pre-strain in the shell, as illustrated in Figure 3c. Since the solidification/drying process may continue toward the inner core even after the fiber has been collected on the roller collector, while a tensile force is applied, a gradient pre-strain field results in the fibers of the final dry membrane. This is a kind of programming, which stores the elastic energy for later on shape recovery [18,31]. However, since the pre-strain is in a gradient manner within the cross-section, during the ethanol wetting-induced shape recovery process, in which wetting starts from the outer-surface toward the inner part gradually, the pre-strained shell contracts (Figure 3c), while the inner core mostly maintains its shape. The result is buckling/crimping of the fiber, as reported in [25]. If the fiber is about uniformly pre-stretched, buckling may occur, in particular during chemically induced shape recovery [32], but the final shape of the fiber should be more or less straight.

**Figure 4:**
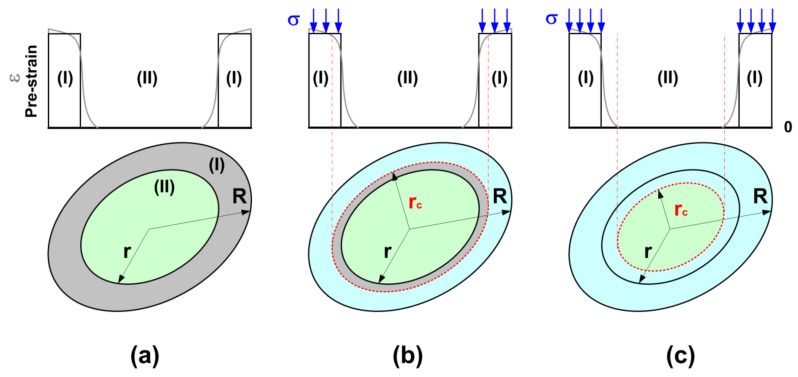
Illustration of cross-section of dried fiber and pre-strain in it. (**a**) Before wetting; (**b**) buckling when the shell is partially wetted; (**c**) buckling when the core is partially wetted.

Refer to Figure 4a for an illustration of the cross-section of the dried fiber (with a radius of *R*) and the pre-strain in it. The process of wetting by ethanol should start from the surface of the dry fiber. For simplification, we may assume that r is the radius of the inner core (layer II, without any pre-strain) and the outer shell (layer I) is uniformly pre-strained.

In the process of ethanol-induced shape recovery, buckling may occur when the critical ethanol penetration depth (*R* − *r_c_*) is in the shell (*R* > *r_c_* > *r*) (Figure 4b) or in the inner core (*r* > *r_c_*) (Figure 4c).

The critical condition for buckling for both cases can be written as [32],

(1) $$F_{cr}=\frac{\pi^{2}EI}{4L^{2}}$$

where *F_cr_* is the critical load for buckling; *L* is the effective length of the fiber, *EI* is the flexural rigidity.

In the case of (*R* > *r_c_* > *r*) (Figure 4b),

(2) $$EI=\frac{\pi }{4}\left[E_{R}\left(R^{4}-r_{c}^{4}\right)+E_{c}\left(r_{c}^{4}-r^{4}\right)+E_{r}r^{4}\right]$$

where *E_R_*, *E_c_*, and *E_r_* are the Young’s modulus of the ethanol softened TPU (pre-strained shell), the Young’s modulus of the pre-strained but yet softened TPU (shell), and the Young’s modulus of the inner core (without pre-strain), respectively.

After wetting by ethanol, the Young’s modulus of TPU MM5520 drops dramatically, and the variation in its Young’s modulus after pre-stretching is limited [33]. Here, we may define *E_h_* and *E_s_* as the Young’s moduli of TPU MM5520 before and after wetting by ethanol, respectively (i.e., *E_r_* = *E_c_*).

Consequently, Equation (2) may be reduced to

(3) $$EI=\frac{\pi }{4}\left[E_{h}r_{c}^{4}+E_{s}\left(R^{4}-r_{c}^{4}\right)\right].$$

The internal compressive force of the wetted/softened part due to the release of elastic energy in the pre-straining part may be estimated by

(4) $$F=\pi \left(R^{2}-r_{c}^{2}\right)\sigma$$

where *σ* is the compressive stress acting on the softened part. Same as in [31], we may assume

(5) $$\sigma =\varepsilon E_{s}$$

where *ε* is the pre-strain.

In the other case of (*r* ≥ *r_c_*) (Figure 4c), the compressive force in the pre-strained shell, i.e.

(6) $$F=\pi \left(R^{2}-r^{2}\right)\sigma$$

is not high enough to induce buckling. Partial softening of the inner core is required. Equation (3) is still valid.

Since many fibers with different diameters (the difference might be only slight) and different effective lengths entangle together in a membrane, some fibers may lose their stability earlier than the others during stimulus-activated shape recovery. The buckling condition may also affect the amount of shrinkage of the membrane. However, for a particular polymer, the most important parameters that affect the amount of shrinkage of a membrane should be the effective length of the fibers, the diameter of the fiber, the thickness of the pre-strained shell, the amount of pre-strain in the shell, and the rotation speed of the roller (in the case of 2D shrinkage). Consequently, these processing parameters, such as the applied voltage, solution concentration, distance between electrodes, rotation speed of collector, and solution flow rate, which can affect the solvent evaporation speed, solidification speed, stretching force, and fiber flight time are important factors. The porous ratio, which is indirectly related to the effective length of the fibers, might be important for the critical buckling condition but less influential on the final shrinkage ratio.

### 3.3. 2D Shrinkage Control

A series of experiments were carried out to reveal the influence of the applied voltage, solution concentration, distance between electrodes, and rotation speed of the collector on the shrinkage of the TPU (MM5520) membrane in the rotational direction and transverse direction (of the roller). The solution flow rate was kept at 1.2 mL/h in these experiments.

**Figure 5:**
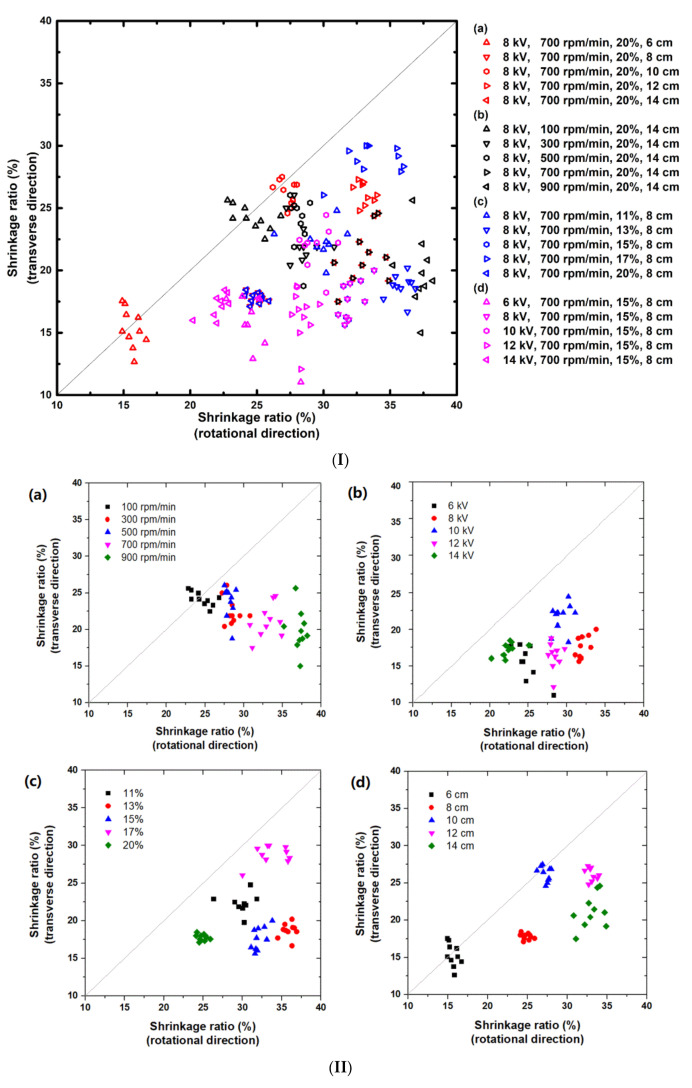
Shrinkage ratio along the rotational direction versus shrinkage ratio along the transverse direction (solution flow rate: 1.2 mL/h). (**I**) Overlapping of all experimental results; (**II**) results of parametric study. (**a**) Rotation speed of collector; (**b**) applied voltage; (**c**) solvent concentration; (**d**) distance between electrodes.

Figure 5I presents the shrinkage ratio in the rotational direction versus that in the transverse direction of all experimental results, which are divided into four groups ((a), (b), (c) and (d)). Refer to the legend for the processing parameters (parameters are labeled in the sequence of applied voltage (kV), rotation speed of the collector (rpm/min), solution concentration (wt %), and distance between electrodes (cm)) of the samples applied in each test. Each test includes at least five samples, which were cut out from the middle part of the same piece of the as-fabricated membrane. Refer to Table S1 in the Supplementary Materials for the processing parameters of each group and the shrinkage ratios of each sample in both the rotational and transverse directions.

Although this parametric investigation is far away from complete, it is found that most of the results are distributed on the lower right of the diagonal, that is, the shrinkage ratio in the rotational direction is greater than that in the transverse direction. This is apparently due to the drafting and alignment effect of the roller drum.

All samples are able to shrink over 10% in both the rotational and transverse directions. The maximum shrinkage in the rotational direction is over 37.5%, while that in the transverse direction is slightly less, about 30%. Below the diagonal, the obtained experimental results cover almost the whole area of 15% to 35% (in the rotational direction) and 15% to 30% (in the transverse direction).

Some results of different groups overlap. Hence, there might be multiple ways to achieve the required shrinkage ratios in both directions.

In Figure 5II, we plot the results of each group separately to reveal the influence of each parameter. Refer to Figure S6 in the Supplementary Materials for the relationship between the average shrinkage ratios in both directions as a function of the parameter that varies in that group of tests.

In Figure 5(IIa), the influence of the rotation speed of the roller is apparent and more or less monotonic. In particular, when the rotation speed is over 500 rpm, the shrinkage ratios change rapidly (refer to Figure S6a in the Supplementary Materials). A higher roller drum speed tends to align fibers better into the direction of rotation (refer to Figures S4 and S5 in the Supplementary Materials) and may cause higher pre-strain in the fibers. Consequently, the shrinkage ratio in the direction of rotation increases with the increase of the rotation speed of the roller, while the shrinkage ratio in the transverse direction reduces but in a lower level (Figure 5a, Figure S6a in the Supplementary Materials). Therefore, the shrinkage ratios in the rotational direction and the transverse direction can be tailored by varying the rotation speed of the roller drum, even in a layer-by-layer manner in the process of membrane fabrication to result in a gradient 2D shrinkage ratio along the thickness direction of the membrane.

Since 700 rpm/min was the applied rotation speed in all other groups, it is expected that the results of those tests should be all underneath the diagonal.

Figure 5(IIa) also shows that with the increase in the rotation speed, the results spread over a wider range. For example, the shrinkage ratio in the transverse direction of 900 rpm/min is from about 15% to 25%.

In Figure 5(IIb), the variable is the applied voltage. According to Figure S6b in the Supplementary Materials, for this particular set of fixed parameters, there is no clear trend for the influence of the applied voltage. This is also found for the influence of the solution concentration in Figure 5(IIc) and Figure S6c in the Supplementary Materials. The applied voltage and solution concentration affect the shrinkage ratio of the membranes through the competition among a number of factors, such as flight time, stretching force, solvent evaporation rate, and solution viscosity. It is interesting to point out that in Figure 5(IIc), the results of 20% solution concentration are within a very small area, which implies that this membrane is very uniform and high quality.

The electric field strength and the flight distance of the fibers vary according to the distance from the nozzle to the collector. In Figure 5(IId), the variable is the distance between electrodes. By varying the distance from 6 to 12 cm, the shrinkage ratio in the rotational direction increases from about 15% to 35% in an approximately linear manner (refer to Figure S6d in the Supplementary Materials). Further increase in the distance to 14 cm does not increase the shrinkage ratio but causes wider spreading of the results and decrease in the shrinkage ratio in the transverse direction. Since the influence of the distance on the shrinkage ratio in the transverse direction is not monotonic, the results of five different distances do not follow any clear trend. The results of some groups (namely 8 cm, 10 cm, and 12 cm) are within a small area, which indicates that the change distance is a more effective way to result in uniform and high-quality membranes.

A surprise spotted in Figure 5(IId) is that the results of two distances, namely 6 cm and 10 cm, are around the diagonal, although the applied rotation speed is 700 rpm/min, while a distance of 8 cm, which is in between, is far away from the diagonal but within a small area.

Although the results reported here are limited, the following findings are apparent:

− There are many processing parameters that affect the shrinkage ratio of the electrospun membranes. Environmental conditions, such as room temperature and humidity, and solution flow rate are not included in this study.
− There are different ways to tailor the shrinkage ratios in both directions.
− Some of the ways are more effective to result in uniform and high-quality membranes.

## 4. Conclusions

In this paper, a gradient pre-strain field is identified as the underlying mechanism for the shrinkage of the electrospun membranes, which is activated by the stimulus-responsive shape memory effect. Crimped fibers in the elecrospun membranes after shrinkage (revealed by SEM) provide solid evidence for buckling, which is associated with the gradient pre-strain field in the cross-section of the fibers. Depending on the electrospun process and material, there are two types of buckling conditions. Due to the nature of the electrospun membrane, the out-shell of the resulted polymeric fibers are pre-stretched, while the inner core is without stretching or less stretched. Hence, shrinkage is more or less an intrinsic feature of all electrospun membranes. Many processing parameters can affect the gradient pre-strain field and therefore the shrinkage ratio of the membranes.

A series of experiments were carried out to reveal the influence of the applied voltage, solution concentration, distance between electrodes, and rotation speed of collector on the shrinkage ratios of a commercial TPU in the rotational direction of the roller and the transverse direction. Although this parametric investigation is far away from complete, it is concluded that there are different ways to achieve the required shrinkage ratios in both directions. Some of them might be more effective to achieve good uniformity.

Via optimization of these parameters, it is possible not only to control the shrinkage but also to realize gradient shrinkage in two in-plane directions along the thickness direction of the membranes.

## Supplementary Materials

The following are available online at https://www.mdpi.com/article/10.3390/mi12080920/s1. Figure S1: Typical SEM images of TPU MM5520 electrospun nanofiber membrane before (a1 and a2) and after (b1 and b2) ethanol infiltration. Figure S2: (a) Photographs of TPU electrospun membranes before (a1) and after (b1) heating to 70 °C. (b) SEM images of the electrospun nanofiber membrane before (a2) and after (b2) the shrinkage. Figure S3: (a) Photographs of poly (lactic acid) (PLA) electrospun membranes before (a1) and after (b1) ethanol infiltration. (b) SEM images of the electrospun nanofiber membrane before (a2) and after (b2) the shrinkage. Figure S4: SEM micrographs of TPU electrospun nanofiber membrane produced at different rotation speeds of the roller collector. Figure S5: Fast Fourier transform (FFT) analysis of Figure S4a,b,d. Directionality plugin of ImageJ software (http://fiji.sc/Fiji (accessed on 1 April 2021), Ashburn, VA, USA) was used to analyze the alignment of fiber. Figure S6: Average shrinkage ratio of electrospun nanofibers membrane produced using different electrospinning parameters. Video S1: Controlled ethanol wetting of membrane. Table S1: Processing parameters and experimental results of shrinkage ratios.
