# Supplementary material for: Stimulus-Responsive Shrinkage in Electrospun Membranes: Fundamentals and Control
Source: Micromachines (Basel). 2021 Jul 31;12(8):920. doi: 10.3390/mi12080920 (PMC8401720; doi:10.3390/mi12080920)
Supplement: Supplementary file 1 [file micromachines-12-00920-s001.zip › Supplementary Materials v2.pdf]

Supplementary Materials

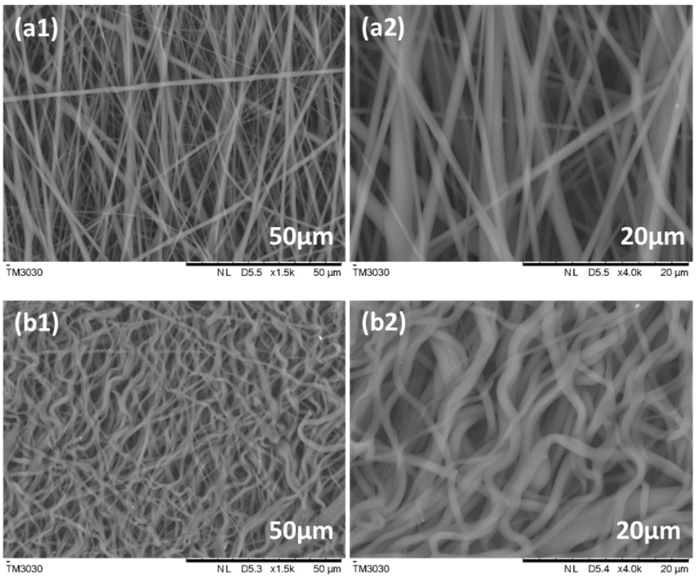

Figure S1. Typical SEM images of TPU MM5520 electrospun nanofiber membrane before (a1 and a2) and after (b1 and b2) ethanol infiltration. The applied voltage, solution concentration, distance between electrodes, rotation speed of collector, and solution flow rate were 8 kV, 15%, 8 cm,700 rpm/min and 1.2 ml/h, respectively.

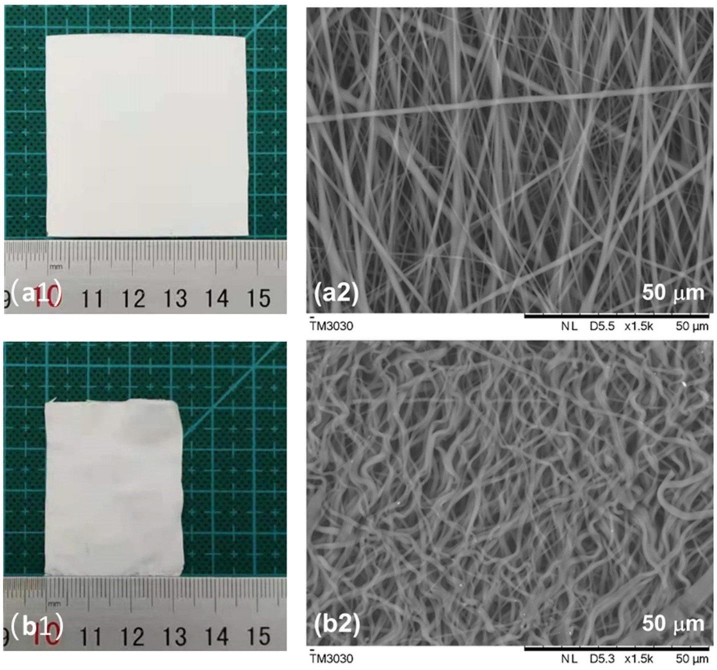

Figure S2 (a) Photographs of TPU electrospun membranes before (a1) and after (b1) heating to 70 ℃. (b) SEM images of the electrospun nanofiber membrane before (a2) and after (b2) the shrinkage. The applied voltage, solution concentration, distance between electrodes, rotation speed of collector, and solution flow rate were 10 kV, 15%, 10 cm,700 rpm/min and 1.2 ml/h, respectively. The TPU was purchased from Hongye Co., Ltd. (Shenzhen, China).

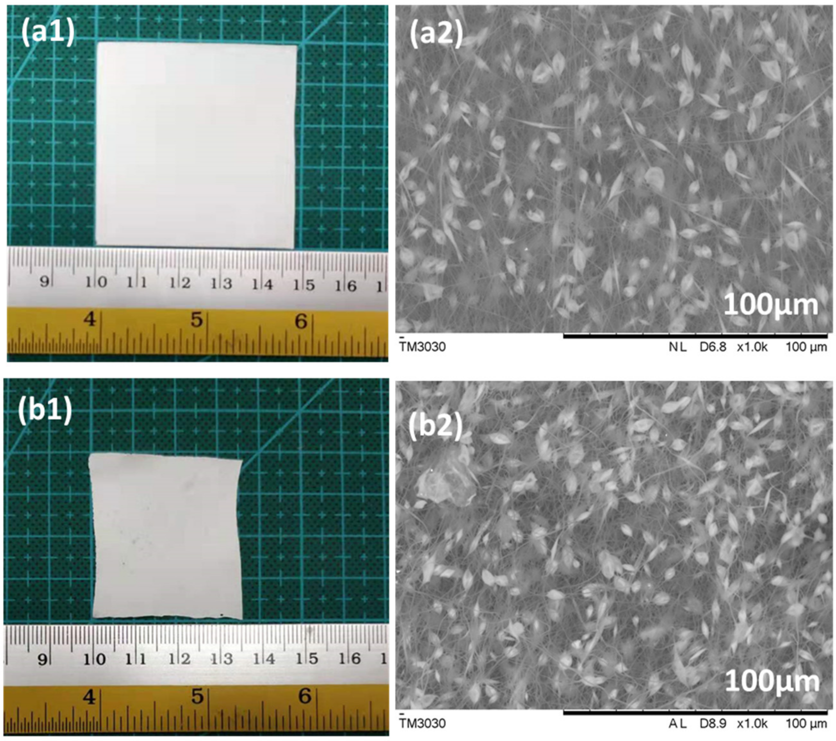

Figure S3 (a) Photographs of poly (lactic acid) (PLA) electrospun membranes before (a1) and after (b1) ethanol infiltration. (b) SEM images of the electrospun nanofiber membrane before (a2) and after (b2) the shrinkage. The applied voltage, solution concentration, distance between electrodes, rotation speed of collector, and solution flow rate were 8 kV, 15%, 8 cm, 900 rpm/min and 1.2 ml/h, repsetively. Poly (lactic acid) (PLA 4060D) was obtained from Nature Works LLC (Minnetonka, USA).

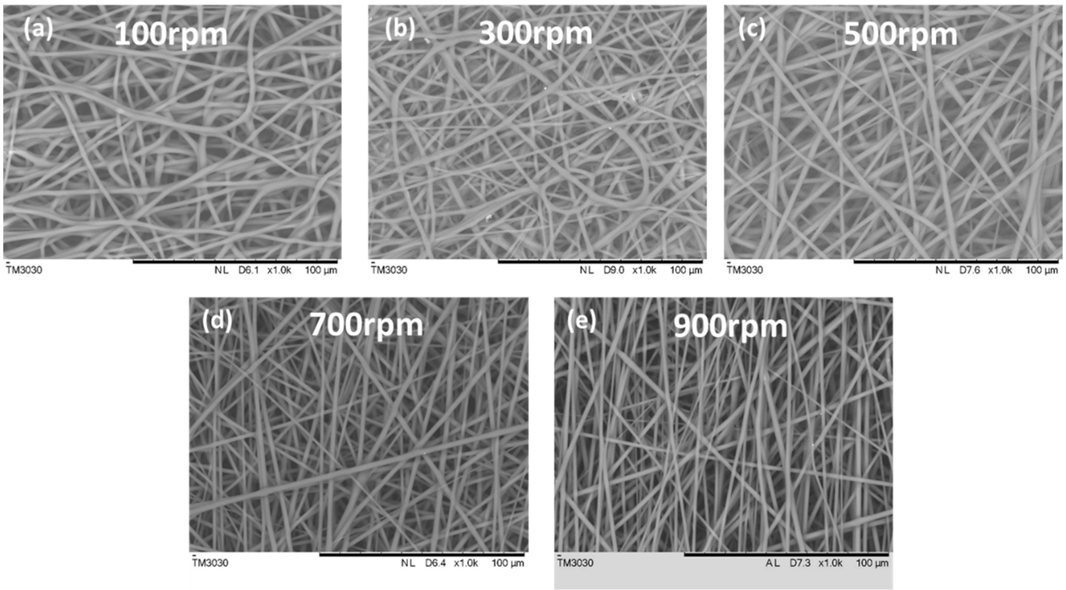

Figure S4 SEM micrographs of TPU electrospun nanofiber membrane produced at different rotation speeds of the roller collector. Refer to Figure 5(a) for the specifics.

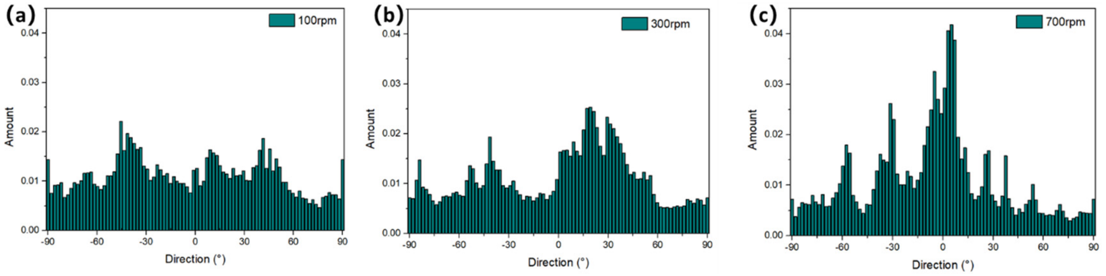

Figure S5 Fast Fourier transform (FFT) analysis of Figures S5(a), (b) and (d). Directionality plugin of ImageJ software (http://fiji.sc/Fiji, Ashburn, VA) was used to analyze the alignment of fiber. A flat histogram is expected for an isotropic distribution, whereas a peak, which corresponds to a preferred orientation, appears in the histogram.

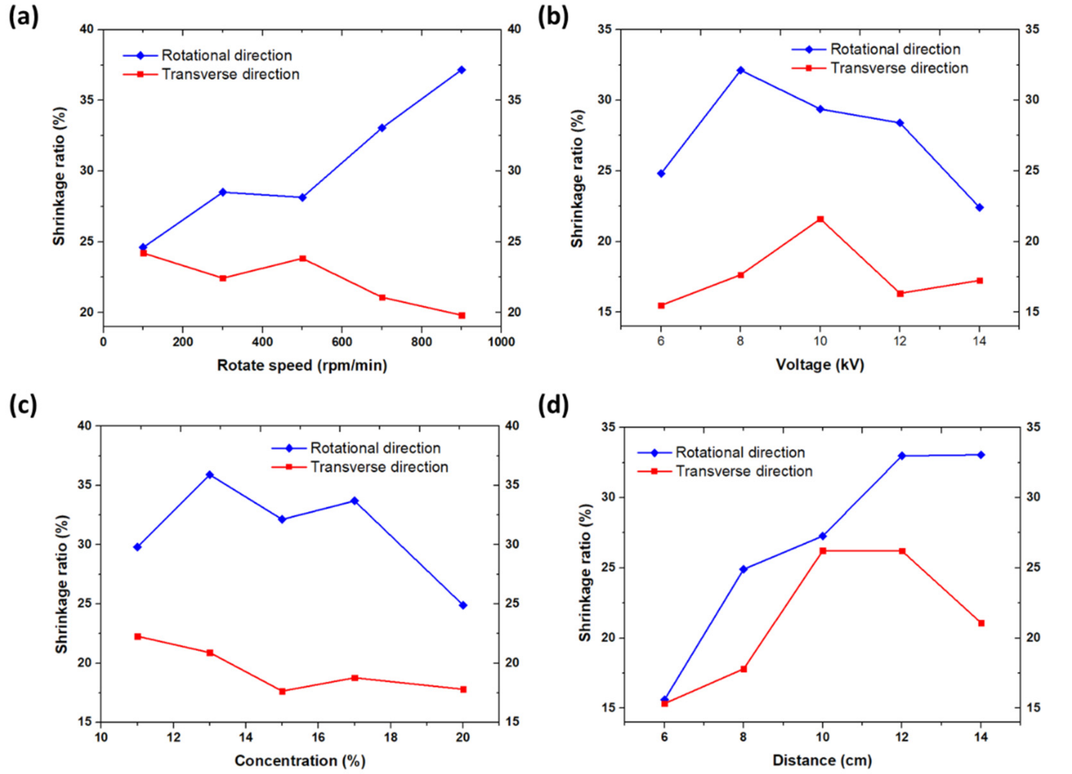

Figure S6 Average shrinkage ratio of electrospun nanofibers membrane produced using different electrospinning parameters. Refer to Figure 5 (a)-(d) for the specifics.

Video S1

Controlled ethanol wetting of membrane.

Table S1 Processing parameters and experimental results of shrinkage ratios.

**Processing parameters** (applied voltage, rotation speed, solvent concentration, distance between electrodes)	**Average shrinkage ratio (%)**	
	Rotational direction	Transverse direction
8 kV, 100 rpm/min, 20%, 14 cm	24.61	24.21
8 kV, 300 rpm/min, 20%, 14 cm	28.51	22.44
8 kV, 500 rpm/min, 20%, 14 cm	28.15	23.83
8 kV, 700 rpm/min, 20%, 14 cm	33.06	21.08
8 kV, 900 rpm/min, 20%, 14 cm	37.17	19.82
6 kV, 700 rpm/min, 15%, 8cm	24.82	15.48
8 kV, 700 rpm/min, 15%, 8cm	32.13	17.65
10 kV, 700 rpm/min, 15%, 8cm	29.38	21.60
12 kV, 700 rpm/min, 15%, 8cm	28.40	16.33
14 kV, 700 rpm/min, 15%, 8cm	22.41	17.24
8 kV, 700 rpm/min, 11%, 8cm	29.79	22.271
8 kV, 700 rpm/min, 13%, 8cm	35.9	20.895
8 kV, 700 rpm/min, 17%, 8cm	33.69	18.771
8 kV, 700 rpm/min, 20%, 8cm	24.9	17.8
8 kV, 700 rpm/min, 20%, 6 cm	15.60	15.33
8 kV, 700 rpm/min, 20%, 10 cm	27.27	26.23
8 kV, 700 rpm/min, 20%, 12 cm	32.98	26.21
